# The Application of Natural Carotenoids in Multiple Fields and Their Encapsulation Technology: A Review

**DOI:** 10.3390/molecules29050967

**Published:** 2024-02-22

**Authors:** Yinglan Li, Yanna Zhao, Huaizhen Zhang, Zhuang Ding, Jun Han

**Affiliations:** 1 Institute of Biopharmaceutical Research, Liaocheng University, Liaocheng 252059, China; a18339882956@163.com (Y.L.); dingzhuang@lcu.edu.cn (Z.D.); junhanmail@163.com (J.H.); 2 School of Geography and Environment, Liaocheng University, Liaocheng 252059, China; zhanghuaizhen@lcu.edu.cn

**Keywords:** carotenoids, encapsulation technology, application, source

## Abstract

Carotenoids, which are inherent pigments occurring in plants and microorganisms, manifest a diverse array of vivid hues. Owing to their multifarious health advantages, carotenoids have engendered substantial interest among scholars and consumers alike. Presently, carotenoids are extensively employed in the realms of food, nutrition and health commodities, pharmaceuticals, and cosmetics, rendering them an indispensable constituent of our quotidian existence. Therefore, the objective of this review is to present a succinct and methodical examination of the sources, constituents, and factors influencing formation of carotenoids. Particular attention will be given to encapsulation strategies that maintain intrinsic characteristics, as the growing desire for carotenoids is propelled by individuals’ escalating standards of living. Moreover, the applications of natural carotenoids in multiple fields, including pharmaceutical, food and feed, as well as cosmetics, are discussed in detail. Finally, this article explores the main challenges hindering the future advancement of carotenoids, aiming at facilitating their effective integration into the circular economy.

## 1. Introduction

Food coloring is derived from either natural pigments found in nature or synthetic pigments. Natural pigments are considered to be more environmentally friendly and beneficial for health when compared to synthetic pigments. Consequently, there has been a growing inclination towards the utilization of natural pigments in diverse industries, as they provide a safer and eco-friendly alternative. Furthermore, natural pigments offer a wide array of colors and possess various health-enhancing properties that contribute to disease prevention and overall well-being [1]. Notably, carotenoids have demonstrated significant potential for advancement in various fields such as cosmetics, nutrition, healthcare, and pharmaceuticals due to their rich nutritional value and healthcare functions [2].

Carotenoids, referred to as tetraterpenoids, are hydrocarbons composed of 40 carbon atoms and two rings at each terminus [3]. They are mainly found in fruits, vegetables, microorganisms, and some marine organisms. A standard carotenoid is comprised of eight isoprene units connected consecutively [4]. These natural pigments are predominantly synthesized by photosynthetic organisms, including plants, algae, and bacteria. While carotenoids are primarily sourced from plants, those derived from microalgal biomass have experienced growing popularity. Given the human body’s inability to internally produce carotenoids, their acquisition is necessary through dietary consumption or supplementation to fulfill physiological needs [5].

In nature, there exists an estimated total of approximately 1100 carotenoids [6], but only a limited number of around 40 are regularly utilized by humans [3]. Among these, β-carotene, lutein, lycopene, and zeaxanthin are commonly consumed dietary carotenoids [7]. Carotenoids can be broadly categorized into two groups: oxygenated xanthophylls (e.g., astaxanthin, lutein, and zeaxanthin) and hydrocarbon carotenes including α-carotene, β-carotene, and lycopene [3]. In recent years, carotenoid-related industries have been gradually developed, resulting in year-on-year increases in profits. It is projected that astaxanthin alone will generate a market share of USD 600 million in North America by the year 2030, indicating promising prospects for growth [8]. 

Research has provided evidence for the noteworthy contribution of carotenoids to the enhancement of human well-being, as β-carotene showcases considerable provitamin A activity [9], lutein and zeaxanthin serve as essential constituents of macular pigment within the ocular system [5], and astaxanthin exhibits formidable antioxidant attributes. The consumption of carotenoid-rich foods has been proven to effectively diminish the occurrence of various chronic ailments, such as cardiovascular disease and cancer [10]. Furthermore, the antioxidant capacity of carotenoids enables the elimination of free radicals, thereby mitigating the probability of cellular oxidative harm and the subsequent development of associated diseases. However, as a result of the existence of a conjugated double bond system and a variable number, carotenoids are susceptible to structural modifications and oxidation [11], rendering their physicochemical characteristics highly unstable and susceptible to external influences such as light, pH, temperature, and so forth. In order to safeguard them from environmental degradation, diverse encapsulation technologies have been utilized to augment the stability and bioavailability of carotenoids [12].

Considering the extensive body of research pertaining to carotenoids, this review aims to provide a comprehensive overview of relevant encapsulation techniques for protecting carotenoids in food substrates. Additionally, it gathers fundamental information on the application of natural carotenoids in multiple real-life fields, including their application in the pharmaceutical field, food and feed field, and cosmetics field. In addition, the sources, compositions, and formation-influencing factors of carotenoids are also addressed, with the goal of improving the understanding of the carotenoid system.

## 2. Sources, Structure, and Influencing Factors of Carotenoids

### 2.1. Sources and Structure of Carotenoids

Carotenoids originate from a diverse range of sources and encompass a category of more than 1100 pigments produced by various phototrophic and non-phototrophic bioorganisms, including algae, photosynthetic microorganisms, plants, and animals [6]. Due to the presence of these pigments, various organisms, such as plants or plant parts, mammals, birds, fish, and butterflies, exhibit vibrant colors. These pigments play a crucial role in the process of photosynthesis and are vital for the proper functioning of photosynthetic organs. Additionally, carotenoids serve as precursors to plant hormones in non-photosynthetic plants, while also serving as photoprotectors and antioxidants [13]. Although humans lack the ability to synthesize carotenoids endogenously, they can fulfill their nutritional needs by obtaining and accumulating these compounds through dietary consumption [14]. Carotenoids are primarily found in fruits and vegetables, but can also be found in molds, yeast, and other sources [15]. Various types of microalgae have been found to contain diverse carotenoids, with *Dunaliella salina* and *Haematococcus pluvialis* being commonly utilized for the production of β-carotene and astaxanthin due to their high carotenoid content [16]. For instance, up to 12% β-carotene (dry cell weight, DCW) is produced from *Dunaliella salina*. For lycopene, the amplest source is tomato, with a content of 45.9 mg/100 g (dry weight, DW).

**Figure 1:**
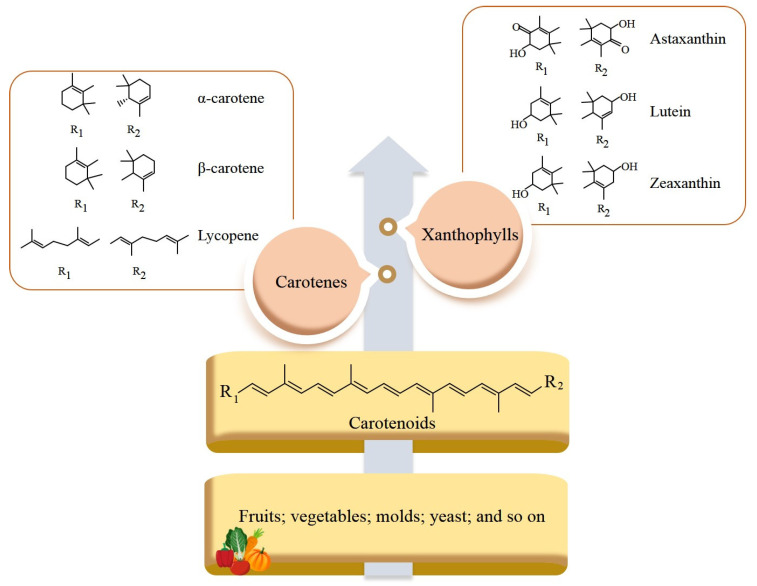
Classification and structure comparison of carotenoids.

Typically, carotenoids consist of 8 isoprene units and 40 carbon atoms, although there are a few advanced carotenoids with 45 or 50 carbon atoms, approximately 40 of which have been identified in certain archaea species [17]. There is a wide range of carotenoids, which can be categorized into two groups based on their structural composition: carotenes and xanthophylls (Figure 1). Carotenes, including α-carotene, β-carotene, and lycopene, encompass only carbon and hydrogen without oxygen. Nevertheless, xanthophylls, which are classified into oxygen-containing carotenoids including lutein, zeaxanthin, and astaxanthin, contain aldehyde, carboxyl, epoxide, and other functional groups. Some xanthophylls can be found in various forms such as glycosides, sulfates, and protein complexes, which contribute to their structural diversity [18]. Furthermore, carotenoids can also be classified as provitamin A carotenoids containing α-carotene and β-carotene, which can be converted into retinol inside the human body, and non-provitamin A carotenoids containing lutein, lycopene, and zeaxanthin, which cannot be transformed into retinol [17].

### 2.2. Factors Influencing Carotenoid Formation

**Figure 2:**
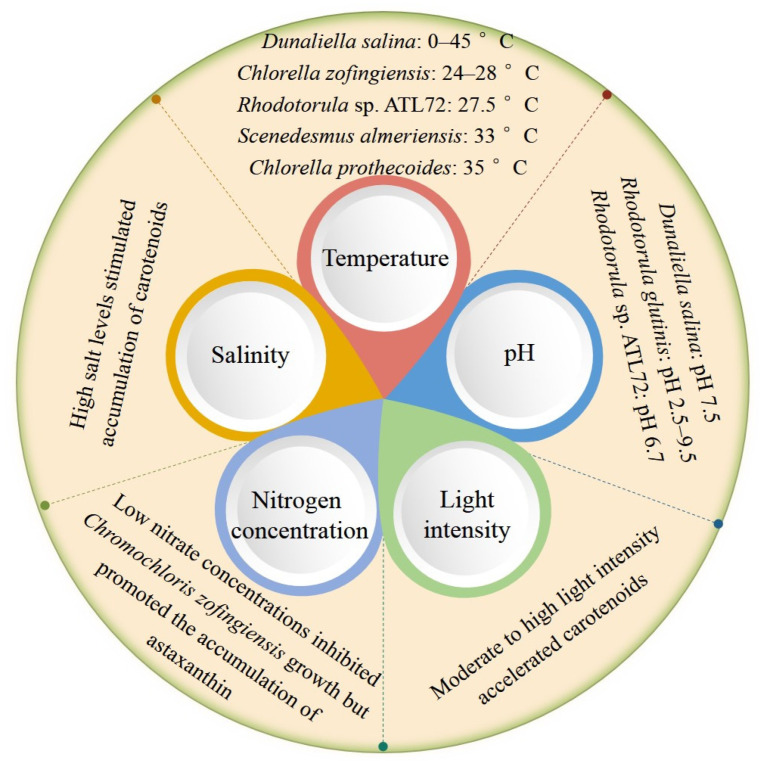
Factors influencing carotenoids formation.

The biosynthesis of carotenoids is a multifaceted and intricate process that encompasses a multitude of physiological and biochemical pathways, subject to regulation by diverse environmental factors including temperature, pH, light intensity, nitrogen concentration, and salinity, among others (Figure 2) [19].

#### 2.2.1. Temperature

In the case of microalgae, temperature exerts a substantial influence on growth, further affecting the biosynthesis of carotenoids. The optimal temperature range for microalgae growth varies across different microalgal species, with the majority exhibiting thriving conditions between 20 and 30 °C. Notably, microalgae of the genus *Dunaliella* spp. prefers temperatures around 32 °C, while *Dunaliella salina* exhibits robust growth within a range of 0 to 45 °C [8,20]. The optimal growth temperature range for *Chlorella zofingiensis* is between 24 °C and 28 °C. Additionally, *Rhodotorula* sp. ATL72 demonstrates peak carotenoid production at 27.5 °C, which subsequently diminishes as temperature increases, aligning with previously reported findings [21].

The effect of temperature on the accumulation of different carotenoids exhibits distinct differences. According to Sánchez et al. (2008), the lutein content of *Scenedesmus almeriensis* reached its maximum at 33 °C, with a dry weight of 0.54% (d.Wt.), when the temperature ranged from 30 °C to 40 °C, while the content of lutein significantly decreased below 20 °C [22]. Shi et al. (2006) reported that the highest lutein content in *Chlorella prothecoides* was observed at 35 °C, which was consistent with other findings [23]. Lutein levels of *Chlorella zofingiensis* exhibit a significant increase with rising temperature within the range of 20–28 °C, peaking at 28 °C. However, at 32 °C, both lutein and the total carotenoid levels of *Chlorella zofingiensis* decline to less than half of their values at 28 °C. Notably, the optimal temperature for lutein accumulation in *Chlorella zofingiensis* is found at 28 °C, while astaxanthin reaches its peak content at 24 °C [24].

#### 2.2.2. pH and Light Intensity

pH also exerts significant influence on the biosynthesis of carotenoids by microalgae, as evidenced by the varying levels of carotenoid production observed under different pH conditions. For instance, *Dunaliella salina* exhibits maximal carotenoid accumulation at a pH of 7.5 [25], while *Rhodotorula glutinis* produces carotenoids across a broad range of pH values (from 2.5 to 9.5) [26]. Additionally, optimal carotenoids synthesis is achieved in *Rhodotorula* sp. ATL72 at a slightly acidic pH of 6.7 [21].

It is widely acknowledged that moderate light exposure plays a crucial role in promoting carotenoid synthesis in plant tissues. Both phytoene synthase and desaturase are activated in response to white light in wild-type *Chlamydomonas reinhardtii*, resulting in rapid upregulation [27]. Moreover, the accumulation of astaxanthin in *Chromochloris zofingiensis* is three times higher under high light intensity (460 μmol photon m^−2^ s^−1^) than under low light intensity (90 μmol photon m^−2^ s^−1^). This can be attributed to the increased photooxidation reaction caused by high light intensity, which activates more oxygen molecules and accelerates carotenoid production [24].

#### 2.2.3. Nitrogen Concentration

Researchers have previously investigated the impact of varying nitrate concentrations on carotenoid levels in *Chromochloris zofingiensis*. Significant reductions in lutein and in total carotenoid content were observed in the culture at low nitrate concentrations of 5 and 10 mM, as cell growth is significantly inhibited in this case. However, increasing the nitrate concentration from 10 mM to 40 mM resulted in a slight reduction in the overall carotenoid content within the cells. Nevertheless, it was observed that the quantity of astaxanthin remained nearly constant during the whole experiment, suggesting that a lack of nitrogen probably promotes the accumulation of astaxanthin and the reduced growth under low-nitrate conditions would be offset [24]. Coulombier et al. (2020) conducted a study to investigate the impact of nitrogen availability on the biomass of *Nephroselmis* spp. and their production of specific metabolites. The researchers employed batch culture and continuous culture in bioreactors with varying nitrogen conditions (nitrogen sufficiency, nitrogen restriction, and nitrogen deficiency) to analyze the effect on carotenoid content. The findings revealed that, as in most advanced chlorophytes, nitrogen sufficiency promoted the biosynthesis of carotenoids in the genus *Nephroselmis* spp., resulting in a high total carotenoid content. However, under nitrogen restriction and deficiency conditions, the total carotenoid content significantly declined. Notably, the change trend of lycopene was consistent with that of total carotenoids, while that of β-carotene and lutein remained stable in spite of the nitrogen availability [28].

#### 2.2.4. Salinity

Environments with high salt levels are found to be beneficial to the accumulation of carotenoids in *Chlorella zofingiensis*. Notably, astaxanthin stimulation is particularly significant in *C. zofingiensis* at a NaCl concentration of 0.2 M, manifesting a cellular carotenoid content surpassing that of pure cultures by more than two-fold and significantly exceeding that of cultures without NaCl [24]. Under conditions of salt stress, the accumulation of carotenoids serves as a protective mechanism for microalgae cells, increasing their chances of survival by acting as an antioxidant. Furthermore, it is important to note that the optimal salt conditions vary among different species of microalgae. For instance, after subjecting *Haematococcus pluvialis* to a NaCl treatment of 1% (0.17 M) for a duration of 10 days, the astaxanthin content increases from 3.53 mg/g to 17.7 mg/g [29]. Similarly, *Chromochloris zofingiensis* CCAP 211/14 demonstrates the ability to tolerate a moderate concentration of NaCl at 100 mM, and the astaxanthin content is significantly increased by a 200 mM NaCl treatment [24].

## 3. Applications of Carotenoids in Multiple Fields

### 3.1. Application of Carotenoids in the Pharmaceutical Field

#### 3.1.1. Application of Carotenoids in the Prevention of Respiratory Diseases

**Figure 3:**
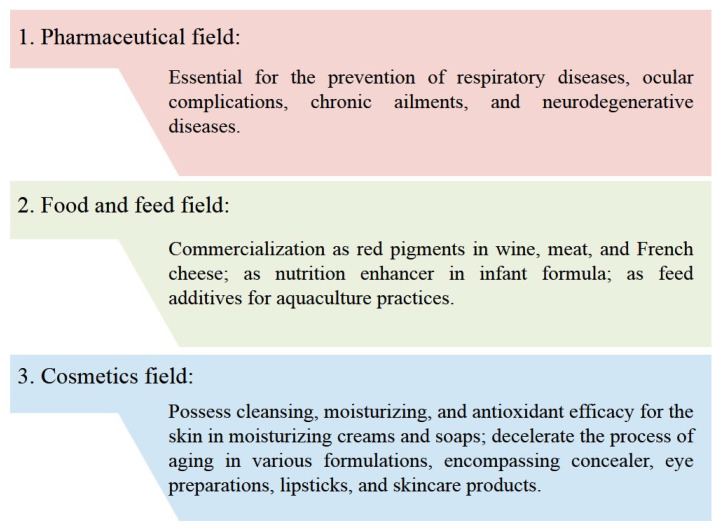
Applications of carotenoids in multiple fields.

The therapeutic attributes of carotenoids, as natural bioactive compounds, render them promising contenders for novel drug exploration and advancement (Figure 3). Respiratory diseases encompass a widespread and intricate array of illnesses. The primary anatomical sites affected by respiratory disease encompass the lungs, chest, trachea, and bronchi. Mild cases frequently manifest symptoms such as a cough and chest pain, whereas severe cases are characterized by dyspnea and respiratory failure leading to fatality [17]. Respiratory diseases not only incur substantial economic losses but also pose a formidable obstacle to prevention and control due to their potential for fatalities and adverse effects on individuals’ quality of life [30].

Over the past few years, the occurrence and fatality rates of chronic obstructive pulmonary disease (COPD) have persisted at elevated levels both domestically and globally, primarily attributable to factors such as air pollution, population aging, and climate change, among others [31]. According to statistical data, COPD has emerged as the third most prevalent cause of mortality globally, resulting in approximately 3 million deaths annually in cases of moderate-to-severe COPD, and this number is showing an upward trend [32]. The prevalence of COPD rises notably with advancing age. Furthermore, the prevalence of diffuse interstitial fibrosis and susceptibility to lung infections in individuals with compromised immune systems has been steadily increasing. Notably, pulmonary infections rank as a leading cause of mortality among individuals afflicted with AIDS. In recent times, numerous global incidents of infectious pneumonia outbreaks have transpired. The substantial mortality rate, potent infectivity, and absence of specific drug interventions have instilled widespread fear and resulted in significant economic repercussions. In contrast, asthma remains the most prevalent chronic ailment, affecting approximately 334 million individuals worldwide, and with an increasing incidence of asthma diagnosis among teenagers [33]. Furthermore, despite successful containment efforts for tuberculosis incidence, there has been a discernible upward trajectory in recent years.

Numerous investigations have substantiated the efficacy of antioxidant vitamins, namely vitamin C, vitamin E, and β-carotene, in augmenting pulmonary function [34]. Additionally, a considerable body of research has established a positive correlation between β-carotene consumption and respiratory health. Nevertheless, the existing literature on the relationship between other carotenoids and lung function remains limited [35]. Grievink et al. (2000) conducted a study in the Netherlands, analyzing blood samples from elderly individuals, and observed that carotenoids present in the blood exerted an influence on pulmonary function [36]. A positive correlation is observed between blood carotenoid levels and respiratory function indices forced expiratory volume 1 s (FEV1%) and forced vital capacity (FVC%) [37], indicating that the consumption of carotenoid-rich foods may confer benefits by improving lung function among the elderly population. Additionally, research has shown that a decrease of one standard deviation in lutein/zeaxanthin levels is associated with a decline in FEV1% and FVC% equivalent to the effects of aging by 1–2 years [34]. It should be noted that no direct correlation between dietary carotenoids and lung function has been definitively established, except for β-carotene. Prominent correlations have been identified among lutein/zeaxanthin and vitamins C and E, as well as FEV1% and FVC% [38]. These investigations substantiate the notion that carotenoids bestow advantageous effects on respiratory well-being. Notably, one contributing factor to this enhanced functionality is directly associated with the water solubility of carotenoids. For example, Dai et al. (2020) prepared an astaxanthin H-aggregates/DNA/chitosan hydrophilic nanocomplex that offered notable advantages, including improved intracellular absorption. Thus, the accumulation of reactive oxygen species in the cytoplasm can be effectively eliminated at the cellular level through increased concentrations [39].

#### 3.1.2. Application of Carotenoids in the Prevention of Ocular Complications

Approximately 50% of the dietary intake of vitamin A is derived from carotenoids, which are abundantly found in a diverse range of fruits, vegetables, and grains. Carotenoids act as precursors to vitamin A, undergo metabolic processes within the body, and exhibit a wide array of therapeutic characteristics. Recent research has revealed that the consumption of carotenoid-rich foods can significantly reduce susceptibility to specific ocular conditions for individuals [40,41,42,43]. Insufficient levels of carotenoids can lead to ocular complications, including dryness, corneal softening, and ulcers, which may ultimately result in permanent vision impairment and other related ailments [44,45]. 

In order to enhance the physicochemical properties and obtain diverse health advantages, researchers have developed lutein-rich nanoemulsions. The water solubility of a nanoemulsion was found to be 726 times higher than that of free lutein, as determined through in vitro and in vivo bioavailability measurements. Moreover, the in vitro bioaccessibility of composite nanoemulsions (87.4%) was significantly greater than that of unencapsulated or free lutein (15%). The in vivo bioavailability of the lutein nanoemulsion (112.6 ng/mL) exhibited a significant increase compared to unencapsulated lutein (48.6 ng/mL) and mixed micelles (68.5 ng/mL). Additionally, the distribution pattern of lutein nanoemulsion in tissues indicated a higher accumulation in the liver and eye compared to the free and mixed micelle feeding groups. These findings indicated that an oleic–linoleic-acid-encapsulated nanoemulsion can effectively improve the bioefficacy of lutein [46].

#### 3.1.3. Application of Carotenoids in the Prevention of Chronic Ailments

Consumption of carotenoid-rich foods or dietary supplements is imperative for the prevention of numerous chronic ailments caused by free radicals, such as cancer [10], diabetes [47], and cardiovascular disease [48]. Carotenoids can serve as secure and efficacious therapeutic agents for mitigating the excessive expression of matrix metalloproteinases in pathological contexts. The metabolite apo-10′-lycopene, derived from lycopene, has been found to confer protection against chronic diseases through the activation of nuclear factor erythroid2-related factor 2 (Nrf2), which in turn induces the expression of phase II metabolic enzymes, leading to the elimination of harmful xenobiotics. Additionally, lycopene and its metabolites exert their influence on hormone metabolism, neuroendocrine differentiation, mitogen-activated protein kinase (MAPK), and protein 53 (p53) signaling pathways, as well as phase II detoxification processes [49], demonstrating their potential in cancer prevention and gene transcription regulation [50]. Consequently, the regular consumption of moderate quantities of tomatoes has been associated with a significant reduction in the risk of developing lung, breast, colorectal, and prostate cancers.

However, lycopene is a highly hydrophobic nutrient with low water solubility and limited bioavailability, which poses challenges to its incorporation into food products. To address this issue, researchers have developed an alginate-based emulsion gel to encapsulate and protect lycopene, which effectively improves the in vitro release and solubility of lycopene and is more conducive to the transportation of lycopene as a nutritional health product in the food industry. In another study, conducted by Yang et al. (2023), pectin was employed as the wall material for the emulsion, which significantly increased the retention rate and bioavailability of carotenoids in citrus juice, thus more effectively exerting their functional food attributes [51].

#### 3.1.4. Application of Carotenoids in the Prevention of Neurodegenerative Diseases

The potent antioxidant properties of carotenoids are attributed to their active components, which have the ability to scavenge free radicals and quench singlet oxygen [52]. It is imperative to comprehend the anti-inflammatory and antioxidant characteristics of different carotenoids and their underlying mechanisms in order to exert a neuroprotective effect, boost immunity, and foster anti-aging effects in both human and animal populations [53]. Lutein inhibits the nuclear factor-κB (NF-κB) signaling pathway, which results in a decrease in the release of lipid peroxidation and proinflammatory cytokines [54]. In Alzheimer’s disease (AD) rat models, lycopene caused an improvement in spatial learning and an impairment of memory, possibly due to the decrease in NF-κB activity and the inhibition of neuroinflammatory cytokine expression [55]. Furthermore, astaxanthin displayed anti-inflammatory and antioxidant properties which further led to depression improvement and cognitive impairments [56]. 

However, most of the carotenoids exhibited poor solubility, which caused great challenges for their delivery across the blood–brain barrier (BBB) in order to realize their neuroprotective characteristics. In recent times, nano-drug delivery systems such as cationic nanoparticles and liposomes have been utilized to enhance the permeability of carotenoids across the BBB [57]. For example, lutein has been loaded into cationic chitosan nanoparticles and administered through the nose for treating AD [58]. The fabricated lutein nanoparticles exhibited homogeneously spherical morphology with a particle size of below 200 nm. In addition, the cationic nanoparticles, with improved antioxidant properties and ROS-generation efficacy, demonstrated a controlled drug release sustained over 96 h and enhanced internalization through the caveolae-mediated endocytosis pathway. Moreover, the bio-distribution of the lutein nanoparticles showed deposits in the brain, verifying their possible use as a treatment for Alzheimer’s.

### 3.2. Application of Carotenoids in the Food and Feed Field

The commercialization of foods containing carotenoids is expanding in Asia. For instance, the fungus Monasacus, which is rich in carotenoids, is employed in rice fermentation to produce red pigments, which are subsequently utilized in wine, meat, and meat byproducts [59]. French cheese contains natural pigments ranging in color from red to orange, which also improve the sensory properties of the cheese. Additionally, lutein-rich infant formula is prevalent in Russia, as it mimics the lutein content found in breast milk and potentially enhances children’s well-being [60].

Because of its instability, poor solubility, and bioavailability, carotenoid encapsulation technology is also employed in functional foods [61]. For example, Ursache et al. (2018) investigated the microencapsulation of carotenoids derived from sea buckthorn, using proteins extracted from whey. The study employed coacervation and lyophilization methods to prepare these microencapsulated carotenoids, which were subsequently incorporated into muffins to enhance their natural color and functional properties. The antioxidant activity and carotenoid content were effectively maintained in the microencapsulated carotenoids, whose addition resulted in an increase in muffin hardness, accompanied by a decrease in elasticity. The sensory analysis indicated that the muffins supplemented with encapsulated carotenoids demonstrated enhanced acceptability due to superior color and comparable flavor to the control group. Although degradation of carotenoids was observed, no significant alteration in color was detected [4]. Medeiros et al. (2019) conducted an evaluation of cantaloupe extracts that are abundant in carotenoids and were encapsulated in gelatin to enhance their solubility in water. These encapsulated carotenoids were subsequently incorporated into yogurt as natural pigments, and an examination was conducted to assess the stability of the pigments. After adding the extract, the yogurt exhibited a yellow hue, with enhanced uniformity and stability [62]. Another investigation, conducted by Himanath et al. (2021), aimed to improve the antioxidant activity and overall quality of yogurt by introducing lycopene nanoemulsions. The mass and antioxidant activity of lycopene were effectively maintained for a duration of 28 days. Furthermore, sensory attributes were also found to be improved in comparison to control samples [63].

In addition, carotenoids such as astaxanthin are also widely used in the field of feed. The current most economically efficient method for producing astaxanthin is through commercial synthesis, which accounts for over 95% of the feed market [64,65]. According to Grand View Research, the global market for astaxanthin was valued at USD 1.0 billion in 2019 and is projected to experience a compound annual growth rate of 16.2% from 2019 to 2027, reaching nearly USD 4.0 billion by 2027. This growth is attributed to the utilization of natural astaxanthin and its various health benefits and safety [65,66]. For instance, astaxanthin demonstrates antioxidant, immune-enhancing, and growth-promoting effects, thereby mitigating oxidative stress and enhancing immune response, disease resistance, and growth performance in various fish and crustaceans. Consequently, the incorporation of astaxanthin in aquaculture practices can bolster the immunity of aquatic organisms, diminish mortality rates, and curtail the misuse of antibiotics [67]. However, the current body of research on the encapsulation of astaxanthin or other carotenoids to optimize feed performance remains limited, necessitating further investigation.

### 3.3. Application of Carotenoids in the Cosmetics Field

Carotenoids are a class of naturally lipophilic compounds that function as natural colorants in diverse domains such as food and skincare [68]. These substances exhibit the ability to bestow vibrant shades upon cosmetics, personal care items, and other goods. Moreover, empirical evidence demonstrates the efficacy of carotenoids in cosmetic formulations. 

For example, research has disclosed that carotenoids have a cleansing and moisturizing effect on the skin, and function as antioxidants to prevent skin damage caused by free radicals from UV rays and other factors. Thus, individuals exhibiting elevated levels of carotenoids in their skin display a youthful appearance relative to their chronological age [69]. In a previous experiment, carotenoids were extracted from palmyrah fruit pulp, dissolved in virgin coconut oil, and then applied in moisturizing creams and soaps. The antioxidant activity of the cosmetic products containing the extract was determined using a DPPH radical scavenging assay, in a comparison against popular commercial creams and soaps. The findings of the study revealed a statistically significant augmentation in the antioxidant activity of cosmetic products containing the palmyra fruit pulp extract [70].

The presence of free radicals and reactive oxygen species, generated by external environmental pollution and the body’s metabolic processes, can lead to detrimental effects on cell membranes and protein structures. Astaxanthin, due to its robust antioxidant properties, demonstrates a nearly ten-fold higher activity compared to other carotenoids, effectively neutralizing free radicals, and safeguarding against various diseases [71].

Furthermore, astaxanthin exhibits the capacity to safeguard proteins against degradation and decelerate the process of aging, thereby establishing itself as a potentially advantageous constituent for cosmetic purposes [72]. In order to improve the delivery efficiency of astaxanthin in cosmetics, silicified liposomes were synthesized through the utilization of tetraethyl orthosilicate within the hydrophilic domain of lecithin vesicles. In addition, the durability of these silicated phospholipids was augmented by their association with certain inorganic or supported lipid bilayer substances, including Boron nitride, a prevalent cosmetic component with characteristics including the distinctive amalgamation of smooth texture, adherence to the skin, and ease of manipulation. The application of astaxanthin liposomes could extend across various formulations, encompassing concealer, eye preparations, lipsticks, and skincare products [73].

## 4. Encapsulation Technology

Functional bioactive compounds have been recognized for their significant health-promoting advantages, specifically in the realms of antioxidant, immunomodulatory, anticancer, and cardiovascular disease prevention. The growing focus on these compounds in recent times can be attributed to their favorable influence on human health [74]. Carotenoids, in particular, have been extensively employed in diverse domains such as nutrition and healthcare, pharmaceuticals, and cosmetics. Despite the various health benefits associated with carotenoids, their practical utilization is constrained by factors such as their susceptibility to physical instability, and degradation when exposed to high temperatures, light, and oxygen [75]. Furthermore, humans are unable to synthesize carotenoids internally and must rely on external intake to supplement the necessary nutrients. The high lipophilicity of carotenoids results in limited absorption within the body. Additionally, the gastrointestinal tract’s capacity for processing carotenoids post-digestion is severely restricted, further contributing to their limited bioaccessibility and bioavailability. Hence, it is crucial to implement precise protective methodologies to safeguard the integrity and stability of their functional attributes, augmenting their effectiveness within in vivo settings, thereby enhancing overall bioavailability [76].

Various delivery systems have emerged to tackle the practical constraints associated with carotenoids, encompassing solubility, stability, bioaccessibility, and bioactivity. Encapsulation is a physicochemical phenomenon wherein one substance is enclosed within another, leading to the formation of particles spanning from nanometers to millimeters in size. While liquids are commonly employed as the encapsulating material, solids or gases can also be utilized to establish a protective physical barrier to micro or nano systems, safeguarding the compound’s stability against potential influences such as oxygen exposure, water vapor intrusion, light penetration, and pH fluctuations [77,78]. The utilization of encapsulation techniques such as microencapsulation and nanoencapsulation has been proven to be a highly efficacious approach for improving the chemical stability of carotenoids, thereby safeguarding their bioactivity throughout diverse processing and storage conditions. The primary motivation behind employing encapsulation is rooted in the inherent instability of these nutrients, which can either possess a limited shelf life or undergo undesirable reactions with other ingredients, resulting in detrimental consequences. Eun et al. (2020) discovered that the encapsulation agent established a protective barrier between the core and wall materials, effectively shielding the compounds from degradation and enhancing the stability of carotenoids [79]. Moreover, encapsulation has the potential to modulate the solubility, interfacial characteristics, and release kinetics of hydrophobic bioactive compounds, thereby enhancing their bioavailability in transport systems, while concurrently influencing their bioaccessibility and absorption (Table 1) [78,80].

Microencapsulation and nanoencapsulation are two encapsulation technologies presently employed in the food industry. The application of microencapsulation to carotenoids can effectively prolong the preservation of nutrients and regulate their release during ingestion or at specific intervals within the gastrointestinal tract. On the other hand, nanoencapsulation serves to safeguard vulnerable bioactive constituents against external influences, thereby enhancing the solubility and bioavailability of challenging-to-absorb active components [86].

### 4.1. Spray-Drying Encapsulation

Spray-drying is a widely employed systematic technique for drying materials, making it the most frequently utilized microencapsulation technology in various industries. During this procedure, very small droplets are produced by spray cap holes with a narrow particle size distribution that are swiftly evaporated by heated air within the drying chamber, leading to the formation of a desiccated end product, which is immediately collected by the surrounding cathode after being charged through the central anode. This approach facilitates the direct transformation of solutions and emulsions into powder or granular forms, generating micrometer-scale particles with ease, rapidity, and cost-effectiveness when compared to alternative methodologies [87].

#### 4.1.1. Emulsions by Spray-Drying

Tucumã oil is rich in carotenoids, yet the chemical instability, easy degradation, and pronounced lipophilic properties of tucumã oil limit its practical application. However, the utilization of spray-drying technology enhances the performance of tucumã oil by improving its storage stability, in vitro digestion, and gradual release of carotenoids within the gastrointestinal tract. Moreover, the product demonstrates stable chemical properties when subjected to food-processing environments at high temperature, suggesting its potential as a functional ingredient in processed foods [81]. Ribeiro et al. (2020) conducted an experiment in which oil-in-water emulsions were prepared using brill oil, soy protein isolate, and high methoxylated pectin as encapsulating materials. These emulsions were then subjected to spray drying with the assistance of maltodextrin as a drying aid, resulting in the production of microcapsules rich in carotenoids with high rates of encapsulation and retention of bioactive compounds [88].

#### 4.1.2. Liposomes by Spray-Drying

Liposomes, characterized as amphiphilic bilayer systems comprising one or more phospholipids, possess several advantageous properties such as non-toxicity, human safety, biocompatibility, biodegradability, and stability [89]. Owing to their structural resemblance to cellular membranes, liposomes can effectively permeate the cell interior through fusion with the cell membrane [90]. Soy lecithin, marine lecithin, and lactate are widely employed phospholipids in the preparation of liposomes [91,92,93]. The hydrophilic nature of the two lipophilic bilayers facilitates the encapsulation of hydrophobic compounds within the core and bilayer, respectively [93,94]. Due to their potential roles in transportation, protection, and controlled release, they can serve as a carrier for lipid-soluble substances that confer health benefits to humans, such as vitamins, flavonoids like quercetin, folic acid, curcumin, betaine, and carotenoids. This utilization enhances the solubility and bioavailability of said compounds [95,96,97,98,99,100]. 

**Figure 4:**
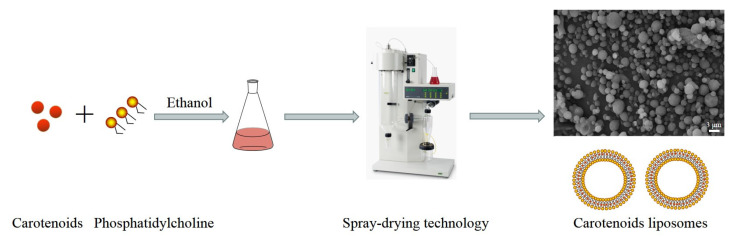
The preparation process of carotenoid liposomes via spray-drying technology. The SEM image was reproduced with permission from ref [82]. Copyright (2013) John Wiley and Sons.

A study conducted by Moraes et al. (2013) investigated the production of liposomes composed of β-carotene, hydrogenated phosphatidylcholine, and sucrose, using spray-drying technology (Figure 4). The study also evaluated the protective capabilities of these liposomes for encapsulated carotenoids under different atmospheric conditions (conventional atmosphere and vacuum), as well as their crystallinity, thermal behavior, solubility, and moisture absorption. The results indicated that liposomal encapsulation of β-carotene demonstrated favorable solubility and a high encapsulation efficiency of 90%. Furthermore, the liposomes exhibited minimal degradation and maintained their original color dispersion after a storage period of 60 days [82].

### 4.2. Lyophilization Encapsulation

The process of lyophilization, also known as freeze-drying, involves the freezing of water-containing materials below their freezing point to convert the water into ice. Subsequently, the ice undergoes sublimation in a vacuum environment, bypassing the liquid phase entirely [101]. The resulting vapor is eliminated through condensation. Thermal radiation is commonly responsible for supplying the necessary heat for vaporization in this process. Briefly, a typical lyophilization process consists of the following steps: A vial containing the solution undergoes crystallization at a low temperature and is placed into a temperature-controlled lyophiliser, whose pressure reduces to a set value. The lyophilization is initiated, and the water vapor is gradually sublimated from the frozen materials.

The utilization of freeze-drying technology offers a notable advantage, as it avoids subjecting food ingredients to high-temperature heating processes. Instead, these ingredients undergo low-temperature sublimation, resulting in the production of high-quality products. Consequently, this technology is highly suitable for preserving unstable ingredients such as pigments, proteins, and yeast [102]. Notably, carotenoids benefit significantly from this encapsulation technique, which has proven successful in fortifying and supplementing food with carotenoids [103]. The encapsulated raw material powder increases the versatility of its application and enables different food substrates to present rich colors. Sharma et al. (2020) discovered that the utilization of freeze-drying technology in packaging carotene extracted from Cucurbita maxima residues enhanced its suitability for direct consumption or solubility in water. Furthermore, the encapsulated carotene could serve as functional components or natural dyes in food [104].

The inherent instability of natural carotenoids, both in terms of their physical and chemical properties, renders them susceptible to degradation and color alteration when expose to various storage conditions. To address these issues, the researchers employed whey protein isolate as an emulsifier to emulsify and encapsulate pequi and buriti oil, which were subsequently freeze-dried. The incorporation of encapsulation techniques has been found to significantly mitigate the loss of carotenoids and enhance their oxidative stability, thereby effectively safeguarding these valuable compounds [83].

### 4.3. Co-Crystallization Encapsulation

Co-crystallization has emerged as a highly regarded encapsulation technique, garnering significant interest from scholars in recent times owing to its uncomplicated operational procedure [105]. In the course of the packaging process, the bioactive component is introduced into a supersaturated sucrose solution, resulting in the formation of crystalline structures that effectively entrap the active substances within the sucrose matrix. The sucrose undergoes a transition from a crystalline arrangement to agglomerated crystals measuring less than 30 μm, characterized by an irregular and porous morphology that affords abundant voids and an augmented surface area for the integration of bioactive substances [106]. Furthermore, the utilization of co-crystallization technology enables the integration of bioactive components into a more enduring powdered form, thereby enhancing the preservation of the enclosed substance to a greater extent. Consequently, co-crystallization technology finds widespread application in both the pharmaceutical and food sectors.

**Figure 5:**
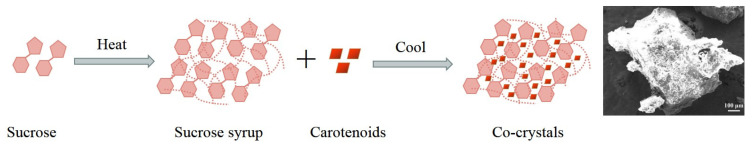
The encapsulation process of co-crystallization for carotenoids within a sucrose matrix. The SEM image was reproduced with permission from ref [84]. Copyright (2021) Elsevier.

Kaur et al. (2021) encapsulated carotenoids within a sucrose matrix using co-crystallization encapsulation technology (Figure 5), and the antioxidant capacity, thermal behavior, and physicochemical properties of the encapsulated carotenoids were also evaluated. The findings demonstrated that co-crystallization with sucrose significantly enhanced the encapsulation efficiency and antioxidant capacity of carotenoids, resulting in levels as high as 77.58% and 68%, respectively. The thermal stability of the carotenoids encapsulated in co-crystals was confirmed through an analysis of DSC thermograms, XRD patterns, and FTIR spectra, while the crystal properties and functional groups of sucrose remained unaltered. Additionally, SEM results revealed distinct irregular cavities within the sucrose cube crystals, providing evidence of the successful encapsulation of carotenoids within the sucrose matrix. Hence, the findings indicated that the inclusion of sucrose in co-crystallization processes substantially improved the overall stability of carotenoids, thereby rendering them suitable for practical applications as natural dyes, sweeteners, and antioxidants in various production scenarios [84].

### 4.4. Hydrogel Encapsulation

Hydrogels are solid and translucent matrices comprising polymeric constituents, and possessing remarkable water absorption and retention properties owing to their intricate three-dimensional network architecture, which facilitates the entrapment of water molecules within their interior [107]. This distinctive network structure enables hydrogels to retain as much as 90% of water, rendering them highly hydrophilic reticular gels [108]. The hydrogel production technology is based on the gelation of fluid phases, which can be achieved through various methods such as temperature adjustment, acidification, crosslinking, or the addition of polyvalent ions [109]. This technology offers effective encapsulation and safeguards hydrophilic and lipophilic bioactive substances from degradation, while also allowing controlled substance release [110,111]. Hydrogel offers sizable scope for tailored function via a three-dimensional structure, which typically traps oil-in-water emulsion droplets in a biopolymer network. The emulsion droplets are fabricated with hydrophilic and lipophilic bioactive substances solubilizing in an oil phase, forming an oil-in-water emulsion with an aqueous phase consisting of a suitable emulsifier via homogenization.

**Figure 6:**
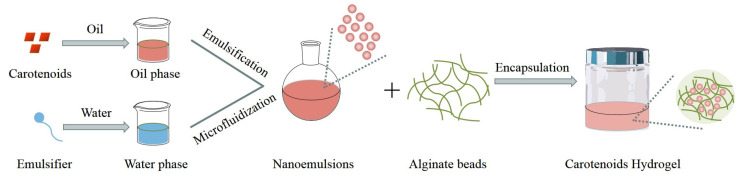
The encapsulation of β-carotene in hydrogel.

Zhang et al. (2016) successfully encapsulated β-carotene in hydrogel without experiencing any degradation. This was achieved by incorporating β-carotene into a nanoemulsion, which was then encapsulated in an alginate saline gel matrix (Figure 6). Furthermore, the researchers effectively showcased, via simulated gastrointestinal experiments, that the digestion rate and extent of β-carotene within hydrogel beads were comparatively lower than that of free β-carotene. These findings imply that hydrogel beads possess commendable efficacy in safeguarding β-carotene against chemical degradation during storage or digestion, thereby exhibiting superior chemical stability and bioavailability [85]. The chitosan-protein isolate complex hydrogels formulated by Hamdi et al. (2020) have exhibited promising potential as a viable option for wound recovery, owing to their capability to regulate carotenoid delivery, accelerate wound healing, and promote angiogenesis [112].

## 5. Future Prospects

Carotenoids are still impeded by a multitude of obstacles in the actual production process [113]. There is no competitive advantage to traditional production processes [114,115]. The handling of fruits and vegetables within households and industries, as well as the industrial processing of citrus juice and canned tomatoes, generates substantial quantities of waste and byproducts, thereby presenting considerable environmental predicaments. However, these challenges can be addressed through the extraction of compounds with economical value from waste materials. Additionally, the recycling of waste and byproducts can create additional revenue streams, thereby improving the economic viability of fruit and vegetable processing [116]. Fruit and vegetable waste not only possesses a high concentration of carotenoids, but also contains other bioactive antioxidants that offer potential health benefits [117,118]. Extensive research has shown that ultrasonic-assisted extraction is a highly efficient technique for recovering significant quantities of commercially important carotenoids from fruit and vegetable waste materials rich in carotenoids. The utilization of supercritical CO_2_ as a solvent for carotenoid extraction presents a promising avenue for environmentally sustainable food production [116,119,120,121]. Therefore, advancements in biotechnology hold the potential to enhance carotenoid yield and reduce processing costs [8]. 

Furthermore, the production of microalgae is costly, and microorganisms are susceptible to decay. The extended duration of culture time, preservation conditions, extraction techniques, and the utilization of analytical techniques to ascertain compound composition may result in the inadvertent loss of certain valuable metabolites. Therefore, it is imperative to develop more gentle cell lysis methods that do not compromise the integrity of carotenoids, as this is crucial for the long-term viability and economic viability of biorefineries. In addition, microalgae resort to extracting chlorophyll from their nitrogen reservoir to sustain the synthesis of critical cellular components necessary for survival in adverse conditions. Regrettably, this process detrimentally impacts the metabolic flux required for cell proliferation and overall biomass productivity, an aspect that has often been disregarded in previous research endeavors [122]. Hence, it is imperative to prioritize the advancement and efficiency of microalgae species and their valuable outputs, including carotenoids and vitamins. The establishment of effective and sustainable microalgae cultivation systems, incorporating the Internet of Things for intelligent microalgae farming, product extraction within the biorefining framework, and sustainable downstream processing, has the potential to create market prospects for microalgal carotene [123].

## 6. Conclusions

Carotenoids, which serve as naturally occurring bioactive pigments, possess significant importance in promoting human health. In addition to being vitamin A precursors, carotenoids play a crucial role in mitigating the risk of various diseases such as cardiovascular disease, cancer, and macular degeneration, while also enhancing cognition and early development. However, due to their susceptibility to processing and storage conditions, carotenoids may experience decline in their nutritional value prior to ingestion. Consequently, encapsulation has emerged as an effective strategy to enhance the dispersibility, stability, and bioavailability of carotenoids. Moreover, the phasing out of synthetic carotenoids due to health concerns has prompted the exploration of natural coloring as a potentially healthier alternative, although the commercialization of naturally derived products significantly trails behind that of their synthetic alternatives.

## Figures and Tables

**Table 1:** Encapsulation technology for carotenoids.

Encapsulation Technique	Major Carotenoids	Wall Material	Encapsulation Efficiency (%)	Particle Size (μm)	Stability	Bioaccessibility	Reference
Spray-drying	Tucumã oil (rich in carotenoids)	Gum Arabic	120 °C inlet air temperature and different oil proportion (g/kg) 100 (g/kg): 90.6 ± 0.3% 200 (g/kg): 74.2 ± 0.3% 300 (g/kg): 44.4 ± 0.3%	0.6~82.2 μm	Spray-dried encapsulation resulted in at least a 142-fold increase in the thermal stability of tucumã oil and a significant increase in oxidative stability and retention after encapsulation.	After complete digestion, the total release of total carotenoids in tucumã oil microparticles reached 64%.	[81]
	β-carotene	Hydrogenated phosphatidylcholine and sucrose	-	1500 nm	More than 90% of the β-carotene was preserved when refrigerated in a vacuum for 60 days. The liposome dispersion maintained its average size, polydispersity index, and zeta potential over 100 days. After 60 days, the degradation of the encapsulated carotene was minimal, and the color of the dispersion was preserved.	-	[82]
Lyophilization	Buriti and pequi oils (rich in carotenoids)	Whey protein isolate (WPI)	-	0.88 ± 0.03~2.33 ± 0.02 μm	Formulations prepared with WPI (heated and unheated) and then freeze-dried enhanced carotenoid protection (48–30% retention after 50 days of storage) and improved the oxidation stability of the oil (OSI 51 and 46 h) compared to unencapsulated materials (30% total carotenoid retention after 32 days of storage, OSI 20.5 h).	-	[83]
Co-crystallization	β-carotene	Sucrose	77.58%	-	Co-crystallization significantly improved the overall stability of carotenoids.	-	[84]
Hydrogels	β-carotene	Alginate	-	0.5% alginate encapsulated: 285 μm 1% alginate encapsulated: 660 μm	The hydrogel beads partially protected β-carotene from chemical degradation, the extent of which depended on alginate levels within the beads.	The bioavailability of β-carotene encapsulated in hydrogels was relatively low. This might be due to some β-carotene molecules remaining in undigested fat droplets within the hydrogel. Second, fewer free fatty acids and monacylglycerol were available to form mixed micelles, reducing intestinal absorption.	[85]

## Data Availability

The data presented in this study are available in article.
